# The interaction of arsenic and *N*-butyl-*N*-(4-hydroxybutyl)nitrosamine on urothelial carcinogenesis in mice

**DOI:** 10.1371/journal.pone.0186214

**Published:** 2017-10-10

**Authors:** Yuan-Chang Dai, Shou-Chieh Wang, Mohammad Mezbahul Haque, Wei-Han Lin, Lei-Chen Lin, Ching-Hsein Chen, Yi-Wen Liu

**Affiliations:** 1 Department of Microbiology, Immunology and Biopharmaceuticals, College of Life Sciences, National Chiayi University, Chiayi, Taiwan; 2 Department of Pathology, Chiayi Christian Hospital, Chiayi, Taiwan; 3 Division of Nephrology, Department of Internal Medicine, Kuang Tien General Hospital, Taichung, Taiwan; 4 Department of Food Science, College of Life Sciences, National Chiayi University, Chiayi, Taiwan; 5 Department of Forestry and Nature Resources, College of Agriculture, National Chiayi University, Chiayi, Taiwan; University of Navarra, SPAIN

## Abstract

The bladder is an important organ for the storage of excreted water and metabolites. If metabolites with carcinogenic characteristics are present in urine, the urothelial lining of the bladder could be damaged and genetically altered. In this study, we analyzed the interaction of arsenic and *N*-butyl-*N*-(4-hydroxybutyl)nitrosamine (BBN) on mouse bladder carcinogenesis. Our previous study found that arsenic affects BBN-altered urothelial enzymatic activity, protein expression, DNA oxidation and global DNA CpG methylation levels. In this study, two mouse models were used. First, after administering a co-treatment of BBN and arsenic for 20 weeks, BBN alone led to a urothelial carcinoma formation of 20%, and arsenic promoted a BBN-induced urothelial carcinoma formation of 10%. The protein expression of GSTM1, GSTO1, NQO1, and p21 did not change by arsenic along with the BBN co-treatment, but the Sp1 expression increased. In the second mouse model, BBN was a pretreatment promoter; arsenic dose-dependently deteriorated BBN-promoted dysplasia by 10% and 40% at 10 ppm and 100 ppm, respectively. Conversely, BBN pretreatment also accelerated arsenic-induced dysplasia by 30%. The urothelial carcinogenic effect reversed after ceasing BBN for a period of 20 weeks. In summary, three conclusions were drawn from this study. The first is the mutual promotion of arsenic and BBN in bladder carcinogenesis. Second, arsenic dosages without bladder carcinogenicity (10 ppm) or with slight carcinogenicity (100 ppm) promote BBN-induced mice bladder cancer progression. Finally, the dysplastic urothelium had reverted to near-normal morphology after ceasing BBN intake for 20 weeks, providing a good suggestion for people who want to quit smoking.

## Introduction

Bladder cancer is the fourth most common type of cancer in the United States (US) among men [1] and is the ninth worldwide [2]. Although it is the 13th lethal cancer disease worldwide [2], its high recurrence impairs patients’ quality of life and causes an enormous economic burden. Therefore, it is important to prevent the development of bladder tumors by elucidating factors involved in their carcinogenesis. One of the common risk factors for bladder cancer is arsenic exposure [3, 4]. Arsenic occurs naturally in minerals and soil and may enter the air and water. Because arsenic cannot be destroyed in the environment, it can only change its form to inorganic or organic arsenic compounds. Besides working in an environment in which arsenic-related materials are used, the common arsenic sources are from food and water [5]. A previous study including 8102 residents in the arseniasis-endemic area of Taiwan indicates that the relative risks of urothelial carcinoma are 1.9-, 8.2-, and 15.3-fold for well water arsenic concentrations of 10.1–50.0, 50.1–100, and >100 μg/liter, respectively, in comparison with those less than 10.0 μg/liter [6].

Other factors of bladder cancer include exposure to cigarette smoking [7], arylamines [8], and meat that contains carcinogens *N*-nitrosamines [9]. One of the *N*-nitrosamines, *N*-butyl-*N*-(4-hydroxybutyl)nitrosamine (BBN), which has carcinogenic potential limited to the urinary bladder [10], has been the most commonly used carcinogen in bladder cancer research [11, 12] and identified as an effective and specific bladder carcinogen in rat and mouse studies [13]. Unlike BBN, some evidence has indicated that arsenic alone induces bladder tumors in rats, but not in wide-type mice [14–16]. Further, in addition to bladder tissue, arsenic induces tumors in other regions of the body in mice and rats [17, 18]; therefore, it may not be an ideal carcinogen specific to bladder cancer in rodent models. Nevertheless, dimethylarsenic acid (DMA) promotes BBN-induced bladder carcinogenesis in a rat study [19], indicating that arsenic plays a promoting role. In a human epidemiological study from the US, no increased risk of bladder cancer was identified for arsenic intake at lower exposures (about 100 μg/day), but the odds ratio (OR) raised to 3.67 for smokers exposed to arsenic for 40 or more years [20]. Because *N*-nitrosamines are also present in cigarette smoke [21], tobacco-specific nitrosamines may prompt arsenic-induced bladder carcinogenesis.

In our previous mouse study, we found that 6 weeks of BBN treatment induced pre-neoplastic damage in bladder tissues and that arsenic changed the damaged state of the bladder [12]. Furthermore, we also found that glutathione *S*-transferase (GST) M1 protein, an important antioxidant enzyme in cells, was down-regulated after BBN treatment [22]. In this study, we aimed to analyze the interaction of arsenic and BBN in bladder carcinogenesis in mice.

## Materials and methods

### Animal treatments

Six-week-old C57BL/6 mice were used in this study and were provided by the National Laboratory Animal Center (Taipei, Taiwan). All animals were maintained at our animal care facility in 12-h/12-h light/dark cycles at ambient temperature (22°C) and 55% relative humidity for one week prior to use. At seven weeks of age, the mice were divided into 6 groups (4–10 mice per group), including control as well as various combinations of BBN and arsenic treatments. The details are illustrated in Figure legends 1 and 4. Chemicals including BBN, DMA (Chem Service) and sodium arsenite (Sigma-Aldrich) were added to the drinking water at final concentrations noted in figure legends respectively. The water was replenished twice weekly and lasted 20–26 weeks. All experiments were approved by the Institutional Animal Care and Use Committee (IACUC) of National Chiayi University (Approval No. 103040). During the experimental period, the care and use of the animals followed the guidelines of National Chiayi University IACUC.

### Bladder protein extraction and pathological evaluation

After chemicals treatment, the mice were euthanized by CO_2_ inhalation and their urinary bladders were removed. Each bladder was cut into two halves; one-half was fixed in 10% neutral formalin, and the other was homogenized in PRO-PREP^TM^ protein extraction solution (iNtRON Biotechnology, Korea) (20 μl/mg tissue) to prepare protein extracts. The bladder tissues in 10% neutral formalin were embedded in paraffin and then cut into 3-μm sections on glass slides. The slides were then stained with hematoxylin and eosin (H&E) for microscopic evaluation by a pathologist. This procedure was performed according to a prior study on the nitrosamine-induced neoplastic progression in urinary bladder of mice [23] and the current WHO classification of human urothelial tumors [24]. The urothelial proliferative/neoplastic lesions of mice were classified as follows:

1. Normal urothelium: **≤** 3 layers of epithelium without appreciable atypia;
2. Hyperplasia: increased urothelial thickness ≥ 5 layers of epithelium without atypia;
3. Flat dysplasia: appreciable cytologic and architectural atypia in flat form;
4. Nodular dysplasia: appreciable cytologic and architectural atypia in nodular growth;
5. Urothelilal carcinoma, superficially invasive: carcinoma invading lamina propria;
6. Urothelial carcinoma, deeply invasive: carcinoma invading muscularis propria or deeper.

### Western blotting

Analytical 10% sodium dodecyl sulfate (SDS)-polyacrylamide slab gel electrophoresis (PAGE) was performed. Thirty μg protein extracts of each sample was analyzed. For immuno-blotting, proteins in the SDS-PAGE gels were transferred to a polyvinylidene difluoride membrane by a trans-blot apparatus. Antibodies against target proteins and β-actin were used as the primary antibodies. Immunoblot analysis was carried out with mouse, rabbit or goat IgG antibodies coupled to horseradish peroxidase. The enhanced chemiluminescence kit and Luminescence Image System (Hansor, Taiwan) were used for detection, and the quantity of each band was determined by the software of MultiGauge.

### Antibodies

Anti-GSTM1 (GTX113448), anti-β-actin (GTX109639) and anti-α-tubulin (GTX112141) antibodies were purchased from GeneTex (Taichung, Taiwan). Anti-NAD(P)H quinone oxidoreductase-1 (NQO1) (ab2346) was purchased from Abcam (Cambridge, UK). Anti-transcription factor Sp1 (Sp1) (07–645) was purchased from Millipore (Billerica, MA, USA). Anti-GSTO1 (15124-1-AP) was purchased from Proteintech (Chicago, IL, USA). Antibodies against p21 (sc-397) and cyclic AMP-dependent transcription factor ATF-2 (ATF2) (sc-6233) were purchased from Santa Cruz (Dallas, Texas, USA). Peroxidase-conjugated secondary antibodies were purchased from Jackson ImmunoResearch (West Grove, PA, USA).

### Statistical analysis

Numerical data are expressed as the mean ± standard error for all samples. Statistical differences were analyzed by Student's t-test. When *p* < 0.05 was considered significant, and * means *p* < 0.05, ** means *p* < 0.01 and *** means *p* < 0.001. All statistics were calculated using SigmaPlot version 11.0 (Systat Software, San Jose, CA, USA).

## Results

### No significant changes in body weight gain after drinking 300 ppm BBN and/or 10 ppm arsenic for 20 weeks

**Fig 1:**
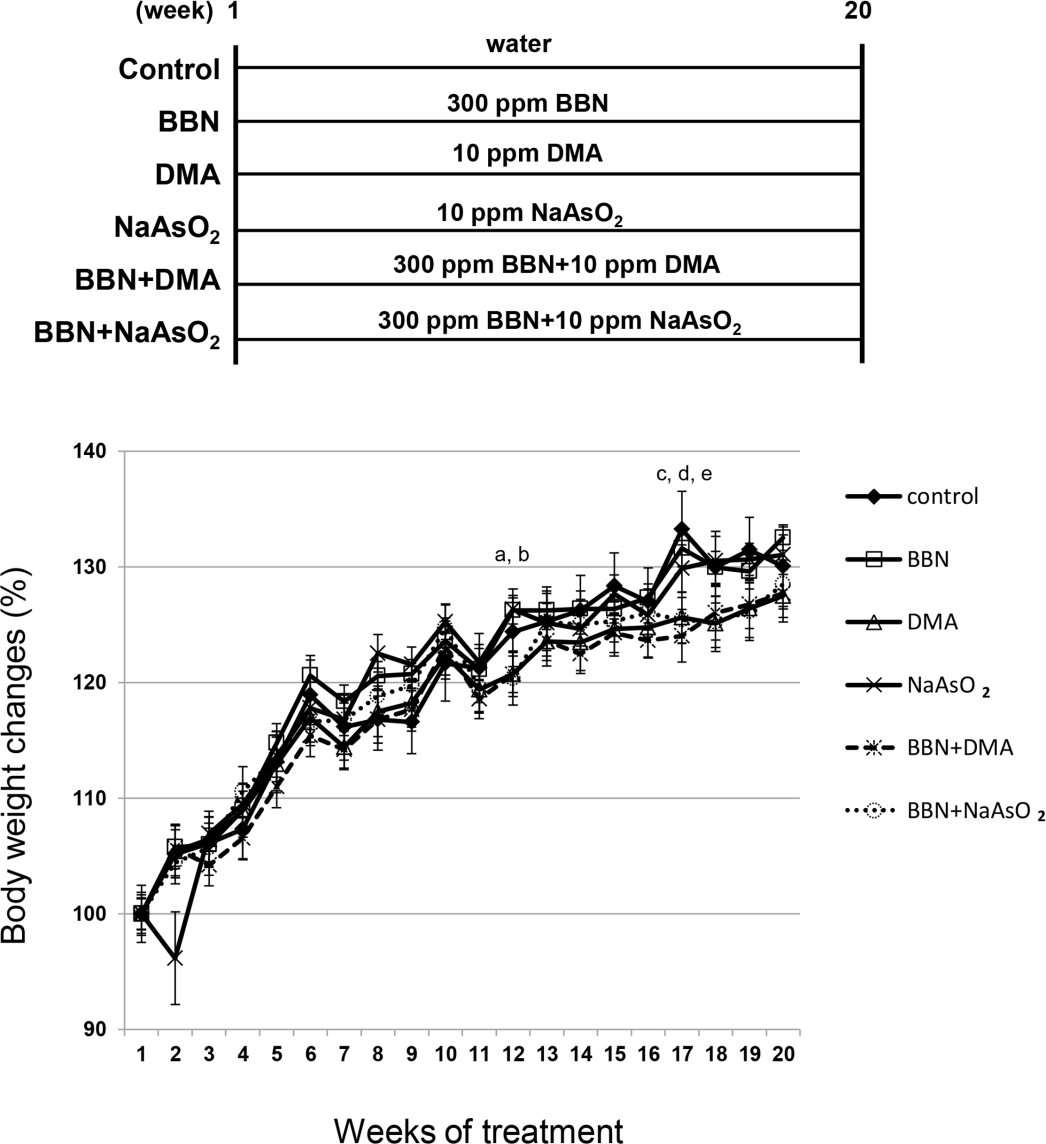
Effects of 300 ppm BBN and 10 ppm arsenic on female mouse body weight. There were 10 mice in each group, 60 mice totally. Weights were recorded once every week. BBN and arsenic were administrated from week 1 to week 20, and mice were euthanized at week 20. After the statistical calculations, two significant differences among the 6 groups were found. One was at week 12, a: NaAsO_2_ vs. control, b: BBN plus NaAsO_2_ vs. control. The other was at week 17, c: DMA vs. control, d: BBN plus DMA vs. control, e: BBN plus NaAsO_2_ vs. control.

The mouse body weight increased gradually throughout the 20-week treatment period. According to the statistical analysis, there were only two different points between the 6 groups, one was at week 12, and the other was at week 17 (Fig 1). During the weeks 17–20, the body weight gain for groups DMA, BBN plus DMA, and BBN plus NaAsO_2_ increased at a slower rate than that of the control group at week 17, but there was no significant difference at week 18, 19 or 20. This finding suggests that the difference at weeks 12 and 17 might not be important. Moreover, using the treatment dosage comprising 300 ppm BBN, 10 ppm DMA, and 10 ppm NaAsO_2_, no significant body weight changes between groups were found at the 20-week end point.

### Pathological evaluation of the mouse bladders following 300 ppm BBN and/or 10 ppm arsenic treatment

**Fig 2:**
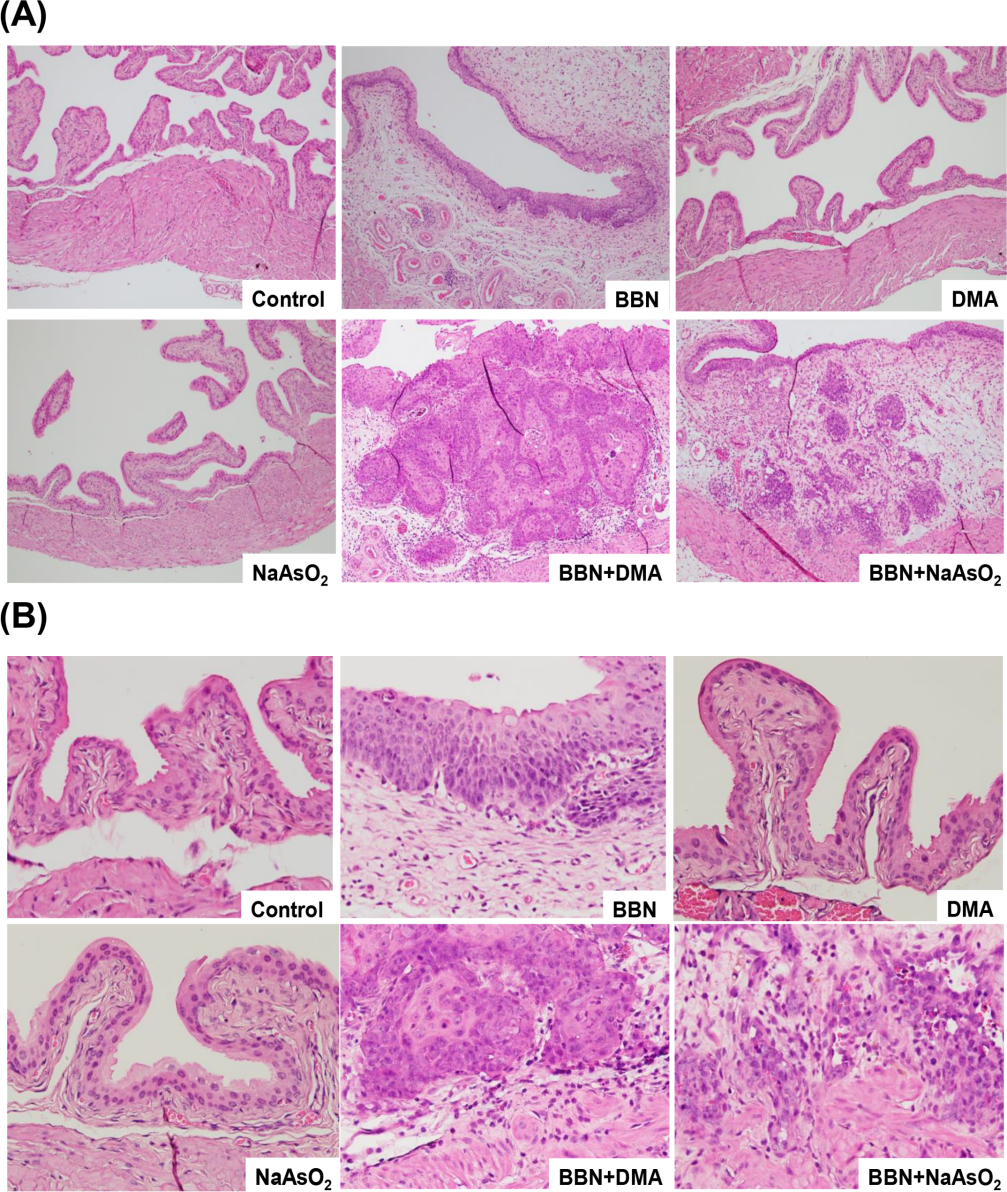
Effects of 300 ppm BBN and/or 10 ppm arsenic on the histopathological changes of mouse bladder tissues. The bladder tissue slides were stained by H&E and then examined under a light microscope at 100× (A) and 400× (B) magnification. The pathological results shown here are normal (control), flat dysplasia (BBN), normal (DMA), normal (NaAsO_2_), urothelial carcinoma, superficially invasive (BBN+DMA), urothelial carcinoma, deeply invasive (BBN+ NaAsO_2_).

After 300 ppm BBN and/or 10 ppm arsenic treatment for 20 weeks, the histological changes in the bladder tissues of the mice were examined. The sub-epithelial connective layer became thickened in the BBN treatment groups including BBN only, BBN plus DMA, and BBN plus NaAsO_2_ (Fig 2A, 100× magnification). In the control group, the urothelium possessed two to three layers in a regular array (Fig 2B, 400× magnification). The cellular layer of the urothelium increased in the BBN-treated groups but remained normal in groups administered the DMA and NaAsO_2_ treatment alone. A histopathological summary for all six groups is shown in Table 1. All control and arsenic-only mice had normal bladder tissues. In the three groups treated with BBN, 70–80% dysplasia was found. The BBN group had a superficially invasive type of urothelial carcinoma of 20%, and it increased to 30% in the group administered BBN plus DMA. Of note, the BBN plus NaAsO_2_ group had a deeply invasive type of urothelial carcinoma of 10%. In summary, this evidence suggests that 10 ppm arsenic slightly promotes the progression of BBN-induced bladder carcinogenesis; however, no evident cancer formation developed with the administering of 10 ppm arsenic alone.

**Table 1:** A histo-pathological summary of mouse bladders after a 20-week treatment.

%	Normal	Hyperplasia	Flat dysplasia	Nodular dysplasia	UC, superficially invasive	UC, deeply invasive
**Control**	**100**	**0**	**0**	**0**	**0**	**0**
**BBN**	**0**	**0**	**50**	**30**	**20**	**0**
**DMA**	**100**	**0**	**0**	**0**	**0**	**0**
**NaAsO_2_**	**100**	**0**	**0**	**0**	**0**	**0**
**BBN +** **DMA**	**0**	**0**	**60**	**10**	**30**	**0**
**BBN +** **NaAsO_2_**	**0**	**0**	**80**	**0**	**10**	**10**

There were 10 mice in each group, 60 mice totally. Water, 300 ppm BBN or 10 ppm arsenic were administrated from week 1 to week 20, and the mice were euthanized at week 20.

### Bladder protein analysis after 300 ppm BBN and/or 10 ppm arsenic treatment

**Fig 3:**
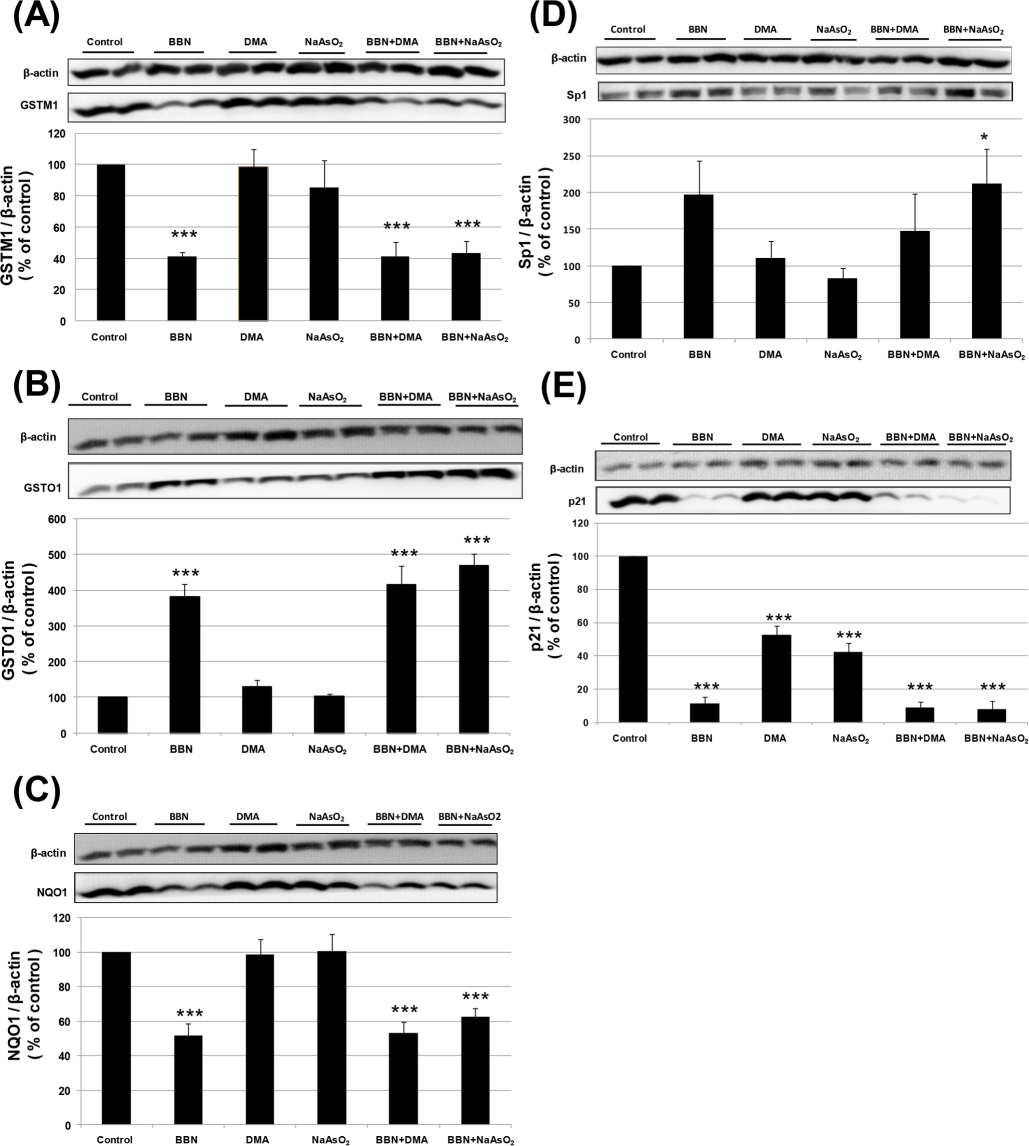
Effects of 300 ppm BBN and/or 10 ppm arsenic on the mouse bladder mucosal protein expression determined by Western blot analysis. Effects of BBN and/or arsenic on the expression of GSTM1 (A), GSTO1 (B), NQO1 (C), Sp1(D), and p21 (E) after a chemical treatment for 20 weeks. The mouse bladder proteins were extracted, electrophoresed and transferred to a membrane. Specific antibodies were used for detection, and beta-actin used as a loading control. The data were quantified from 10 (GSTM1), 6 (NQO1, Sp1) and 4 (GSTO1, p21) samples.

In addition to a histopathological examination, the bladder tissues were analyzed for the expression of proteins associated with tumor development. As indicated in Fig 3A, we found that the GSTM1 protein expression decreased with BBN alone, but not with arsenic alone. On the contrary, BBN alone increased GSTO1 protein expression that has been previously reported (22), but no change was found in the arsenic alone groups (Fig 3B). The expression of another antioxidant enzyme, NQO1, also decreased with BBN alone but remained unchanged with arsenic alone (Fig 3C). The transcription factor Sp1 was slightly increased by BBN alone but did not have a significant difference, as compared with the control group. However, if combined with sodium arsenite, Sp1 protein was increased significantly (Fig 3D). On the other hand, the tumor suppressor gene p21 protein was decreased significantly with administering BBN, DMA or sodium arsenite alone. Although arsenic appeared to slightly diminish BBN-decreased p21 expression, no significant difference existed as compared with BBN alone (Fig 3E). In summary, the protein expression of GSTM1, GSTO1, NQO1 and p21 was affected mainly by BBN, and among them, p21 was also decreased by arsenic alone. Nevertheless, the Sp1 protein expression was significantly elevated with the combined treatment of BBN and sodium arsenite.

### The mouse body weight significantly decreased after drinking 100 ppm arsenic

**Fig 4:**
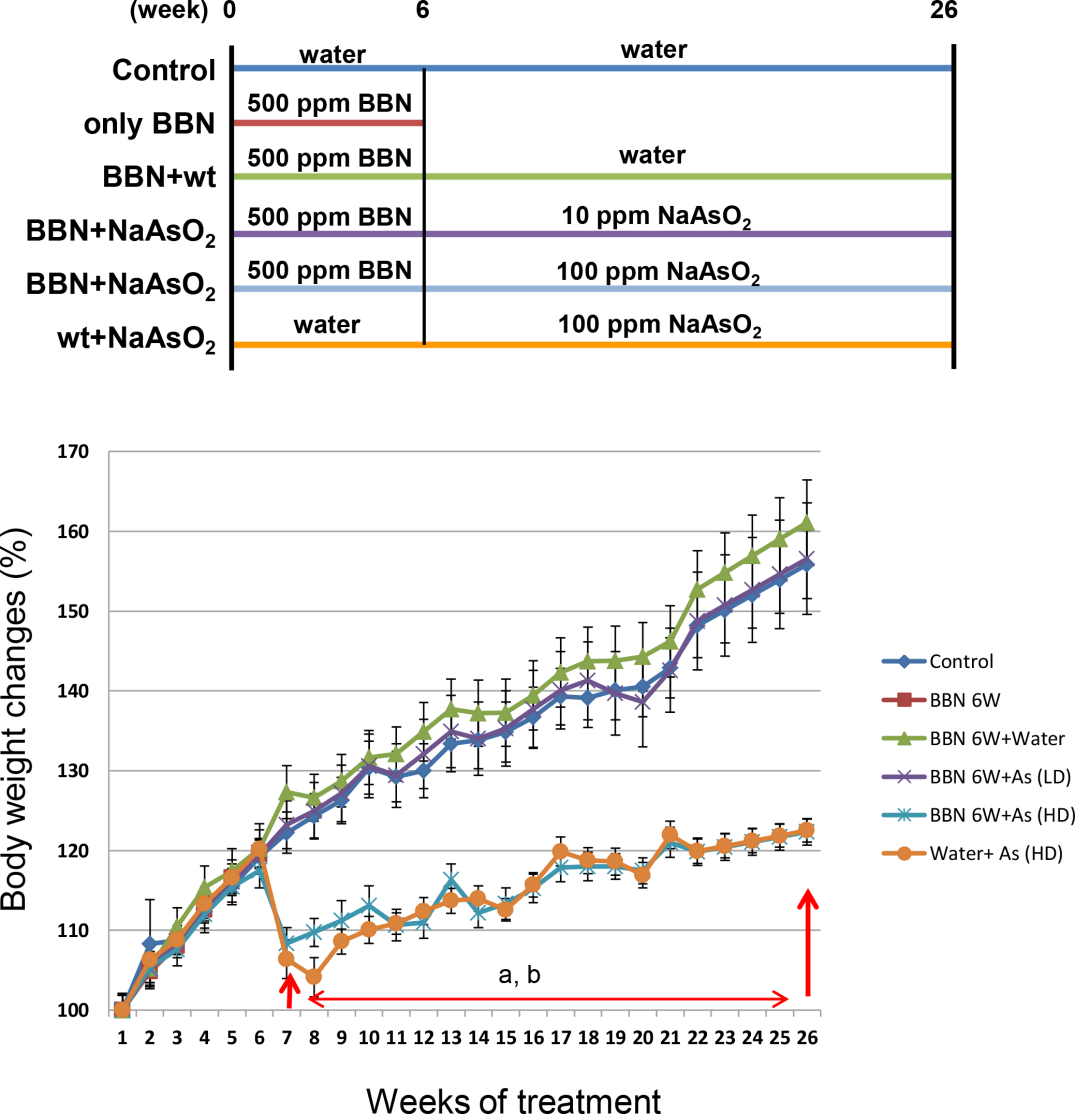
Effects of 500 ppm BBN pretreatment and/or 10 ppm or 100 ppm arsenic on male mouse body weight. There were 10 mice in each group except for 4 mice in group 2 (only BBN, or named BBN 6W). Weights were recorded once every week. Five hundred ppm BBN was administrated from week 1 to week 6, and sodium arsenite (10 ppm or 100 ppm arsenic) was administrated from week 7 to week 26. Mice were euthanized at week 26. After the statistical calculations, significant differences (*p* < 0.01) between the control group and arsenic-treated group were found from week 7 to week 26, including a: 500 ppm for 6 weeks and 100 ppm NaAsO_2_ for 20 weeks (blue line) vs. control (indigo line), b: water for 6 weeks and 100 ppm NaAsO_2_ for 20 weeks (orange line) vs. control (indigo line).

Based on the results of the above mentioned mouse experiments, the effect of arsenic was not obvious with the concurrent BBN treatment. Therefore, to focus more on the effect of arsenic, the mice were pretreated with or without 500 ppm BBN for 6 weeks, followed by treated water only, 10 ppm or 100 ppm arsenic alone for another 20 weeks (Fig 4). During the 500 ppm BBN pretreatment period, no conspicuous body weight changes were discerned between the water and BBN-treated groups. After administering 100 ppm arsenic for 1 week, a significant decrease in mouse body weight was noted, and this trend remained during the 20-week arsenic treatment period (Fig 4). Weight loss indicates that 100 ppm arsenic may influence the appetite of mice.

### Mutual promotion effect of sodium arsenite and BBN on bladder carcinogenesis

**Fig 5:**
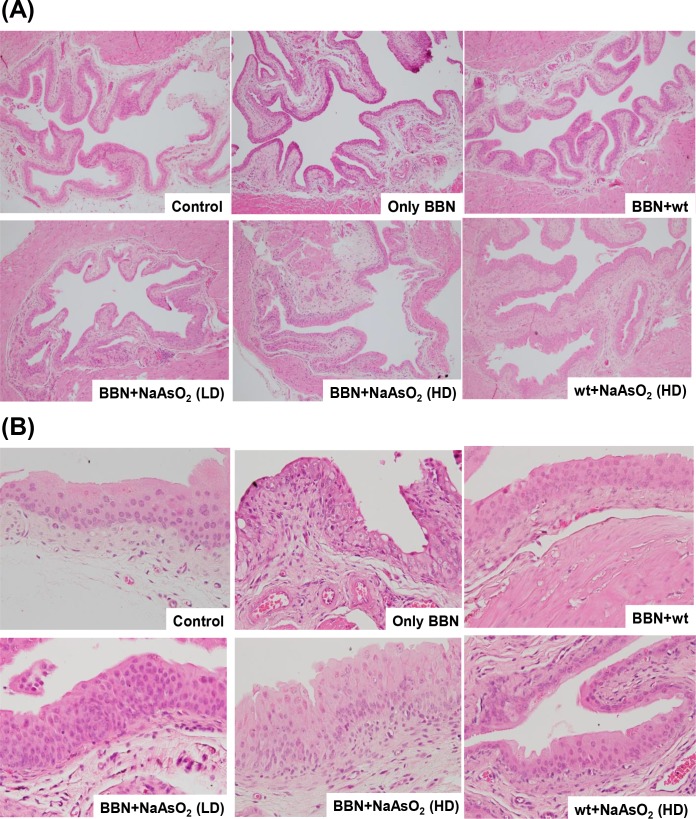
Effects of 500 ppm BBN pretreatment and/or 10 ppm or 100 ppm arsenic on the histopathological changes of mouse bladder tissues. The bladder tissue slides were stained by H&E and then examined under a light microscope at 100× (A) and 400× (B) magnification. (A) The pathological results shown here are normal (control), flat dysplasia (only BBN), normal (BBN+wt), hyperplasia (BBN+10 ppm NaAsO_2_), flat dysplasia (BBN+100 ppm NaAsO_2_), and hyperplasia (wt+100 ppm NaAsO_2_). (B) The pathological results are normal (control), flat dysplasia (only BBN), hyperplasia (BBN+wt), hyperplasia (BBN+10 ppm NaAsO_2_), hyperplasia (BBN+100 ppm NaAsO_2_), and normal (wt+100 ppm NaAsO_2_).

After 500 ppm BBN pretreatment for 6 weeks, followed by water, 10 ppm or 100 ppm arsenic treatment for 20 weeks, the histological changes in the bladder tissues of the mice were evaluated. For the first 6 weeks, the BBN pretreatment caused urothelial dysplasia, loss of umbrella cell phenotype, subepithelial edema, and increased vascularity (Fig 5A and 5B). When the BBN administering was ceased and normal drinking was maintained for the subsequent 20 weeks, the bladder histology was mostly restored and resembled the control urothelium (BBN+wt vs. control in Table 2), and the GSTM1 expression level that had decreased with BBN had also reverted (S1A Fig). This suggests that after ceasing carcinogen BBN intake, the initiated dysplastic changes on the urothelium may have the opportunity to revert to a near-normal morphology. When water was replaced by sodium arsenite, 10 ppm arsenic slightly promoted the urothelium to switch to a flat dysplasia of 10% (BBN+10 ppm arsenic vs. BBN+wt). Furthermore, one hundred ppm arsenic had a higher pontential to induce dysplasia than 10 ppm arsenic did (from 10% to 40%). On the other hand, the BBN pretreatment also increased arsenic-induced dysplasia by 30% (BBN+100 ppm arsenic vs. wt+100 ppm arsenic). Finally, no urothelial carcinoma was found in any group, indicating that long-term treatment with BBN, rather than arsenic, is more critical to cancer development in the mouse bladder (Tables 1 and 2). Even though 100 ppm arsenic alone did not lead to carcinoma, it still induced 10% hyperplasia and 10% dysplasia (wt+100 ppm arsenic vs. control in Table 2). In summary, although high-dose arsenic alone slowly induced urothelial carcinogenesis in mice, it accelerated the bladder carcinogenic effect of BBN pretreatment in mice and vice versa.

**Table 2:** A histo-pathological summary of mouse bladders after a 6-week BBN pretreatment and 20-week arsenic treatment.

%	Normal	Hyperplasia	Flat dysplasia	Nodular dysplasia	Urothelial carcinoma
**Control**	**60**	**40**	**0**	**0**	**0**
**Only BBN**	**25**	**0**	**75**	**0**	**0**
**BBN+wt**	**50**	**50**	**0**	**0**	**0**
**BBN+** **10 ppm** **NaAsO_2_**	**40**	**50**	**10**	**0**	**0**
**BBN +** **100 ppm** **NaAsO_2_**	**30**	**30**	**40**	**0**	**0**
**wt +** **100 ppm** **NaAsO_2_**	**40**	**50**	**10**	**0**	**0**

There were 10 mice in each group except for 4 mice in group 2 (only BBN). Water or 500 ppm BBN was administrated from week 1 to week 6, and water or sodium arsenite (10 ppm or 100 ppm arsenic) was administrated from week 7 to week 26. The mice were euthanized at week 26.

## Discussion

In a previous rat model study, DMA (10–100 ppm) has been proven to promote BBN-induced bladder carcinogenesis in a dose-dependent manner, but no effect was found using 2 ppm DMA [19]. From another rat study, DMA alone induces bladder tumors at dosages higher than 40 ppm, and no evidence of tumors was revealed with dosages lower than 10 ppm [15]. These studies suggest that arsenic alone induces bladder tumors only with a dosage higher than 40 ppm in rats, and it takes about 2 years. In contrast, arsenic induces tumor formation in other body parts, rather than the urinary bladder of mice [25, 26]. In this study, at a dosage of 10 ppm, neither inorganic arsenic nor DMA alone induced urothelial abnormalitie with a 20-week treatment (Table 1), but it slightly promoted (about 10%) tumor progression with the co-treatment of carcinogen BBN (Table 1). In addition, as BBN was a pretreatment inducer instead of a co-treatment carcinogen, both of 10 ppm and 100 ppm arsenic could deteriorate bladder carcinogenesis in BBN-pretreated mice, and 100 ppm arsenic alone also induced 10% dysplasia (Table 2). Our study is the first report to provide information on the promotion effect of arsenic in BBN-induced bladder carcinogenesis in a mouse model.

According to the chemical contamination rules of the US Environmental Protection Agency, the concentration of arsenic in drinking water should not exceed 0.01 ppm, which is only 1/1000 to 1/10000 fold the dosage we used in this animal model. Therefore, a 10- and 100-ppm exposure in this study was a very large amount, as compared with the standard threshold for a human. In fact, some people in contaminated areas have been exposed to arsenic level as high as 5–8 ppm [27]. Remarkably, the health threat has been reported in certain circumstances with a high-level arsenic exposure. For example, a report in Taiwan indicates that the higher the arsenic concentration (0.01–0.05 ppm, 0.05–0.1 ppm, more than 0.1 ppm) in drinking water is, the greater the relative risk of developing bladder cancer in humans [6]. In terms of this mouse study, as 10 ppm or 100 ppm arsenic alone did not lead to urothelial cancer formation after 20 weeks of arsenic intake, it suggests that bladder tumors may not develop easily in humans exposed to arsenic dosages of less than 10 ppm alone. Nonetheless, arsenic has the potential to promote BBN-induced bladder carcinogenesis in mice, and therefore, in humans, the induction of urothelial cancer in areas with arsenic contamination may be accompanied by exposure to other carcinogen(s), for example, cigarette smoking or *N*-nitrosamines intake. In the same concept, an epidemiologic study in Taiwan reported a significantly increased risk of urothelial carcinoma in subjects with high urinary smoking metabolites and total arsenic [28]. Tobacco smoking is a well-known risk factor for bladder cancer [20]; however, arsenic might further promote the effects of bladder carcinogens.

Some reports suggest various mechanisms of arsenic-induced carcinogenesis [5, 29]. For example, some studies have proven that arsenic induces oxidative stress and DNA damage. Arsenic increases DNA 8-hydroxy-2'-deoxyguanosine (8-OHdG) levels in mouse urothelium [12, 30] as well as enhances oxidative stress and 8-OHdG levels in the mitochondria DNA of keratinocytes [31]. BBN also increases DNA 8-OHdG levels and arsenic has an additional effect on mouse urothelium [12]. In addition, arsenic influences gene expression by reprograming DNA methylation. In arsenic-exposed newborns and children, promoter hypomethylation and increased expression of three inflammatory genes was found [32]. Two other reports suggest that arsenic reduces whole DNA 5-methylcytosine methylation levels in the bladders [12, 33]. In contrast, one report indicates that an increased arsenic exposure is associated with a lower gene expression and hypermethylation in human peripheral blood [34]. Because the reprogrammed DNA methylation would induce gene expression change, the effect of arsenic on the DNA methylation of mouse urothelium may be one of the causes contributing to the promotion of BBN-induced carcinogenesis. Furthermore, Sp1, an oncogenic transcription factor, may also be involved in the promotional effect of arsenic [12].

It is known that BBN changes the protein expression of GSTM1, GSTO1 and NQO1 in the mouse urothelium after a 20-week treatment [22, 35]. In this study, BBN also changed their expression, but arsenic did not alter this effect. GSTM1, an antioxidant enzyme related to bladder cancer development [36], is down-regulated in protein and mRNA levels by BBN that are partially mediated by DNA methylation [35]. The decrease in GSTM1 may be involved in BBN-induced bladder carcinogenesis, but not directly related to arsenic. The tumor suppressor gene p21 protein expression was decreased by either BBN or arsenic alone (Fig 3E). It is known that the decreased p21 expression by BBN is not mediated by transcription inhibition in mouse bladders [35]. However, in mouse JB6 epidermal skin cells, arsenic decreases p21 expression through the inhibition of p53 phosphorylation and transactivation [37]. In addition, sodium arsenite also inhibits p21 mRNA expression and adipocytic differentiation in mouse C3H 10T1/2 cells [38]. Thus, the downregulation of p21 by BBN and arsenic may play a carcinogenic effect, whether it is mediated by transcriptional inhibition. To sum up, our data may provide a scenario of bladder carcinogenesis in a mouse model that results from the sequential accumulation of epigenetic, genetic, and post-transcriptional alterations through the effects of BBN and arsenic. Future work would be necessary to elucidate the underlying mechanisms, particularly the carcinogenic effect related to GSTM1 and p21.

In addition to prove the mutual promotion effect of arsenic and BBN in urothelial carcinogenesis, other important information is shown in Table 2. Comparing group 2 (only BBN treatment for 6 weeks) with group 3 (BBN for the first 6 weeks and water for the next 20 weeks), the carcinogenic effect of BBN seemed to be reversed after stopping treatment for 20 weeks. This suggests that once the nitrosamines were ceased, the genomic repair system within the mouse urothelium might reverse the DNA changes. Because *N*-nitrosamines are also present in cigarette smoke [21], a report from Spain suggests that compared with nonsmokers, current smokers (men: OR 7.4; women: OR 5.1) have higher risk of bladder cancer than former smokers do (men: OR 3.8; women: OR 1.8) [39]. In addition, another report concludes that smoking fewer cigarettes over a long time appears to be more harmful than smoking more cigarettes over a shorter time, for equal total pack-years of cigarettes smoked [40]. Recently, a report indicates that cigarette smoking also alters genome-wide methylation that may be related to causing diseases such as cancers, osteoporosis, lung and cardiovascular disorders [41]. Methylation levels of most CpGs returned toward that of never smokers within 5 years of smoking cessation [41]; therefore, the DNA methylation status may have played a role in urothelial reversion after ceasing BBN intake in this study. Despite the carcinogenic effect on the bladder slowed after ceasing BBN intake (Table 2), the urothelial ultrastructure was still slightly altered under the observation of scanning electron microscopy (S1B Fig, BBN+wt vs. control), which was not easily recognized with a light microscopic examination alone (Fig 5A and 5B, S1C Fig).

Based on the evidence revealed in our study, three conclusions were drawn for the first time to our knowledge: The first is the mutual promotion effect of arsenic and BBN in bladder carcinogenesis. Second, arsenic, even at the dosage (10 ppm) without obvious carcinogenicity, also promotes BBN-induced bladder cancer progression in mice. And finally, dysplastic urothelium could revert to near-normal morphology after ceasing BBN intake in mice.

## Supporting information

S1 Fig Effects of 300 ppm BBN pretreatment and/or 10 ppm arsenic on the protein expression. There were 4 female mice in each group, 2 for protein analysis and 2 for morphology analysis. The bladder tissues were homogenized in protein lysis buffer and proteins were extracted for Western blot (2 mice/group)
(PPTX)

S2 Fig Effects of 300 ppm BBN pretreatment and/or 10 ppm arsenic on bladder morphology. Bladder tissues (2 in each group) were filled with fixation buffer and then cut into two halves. One-half was prepared for scanning electron microscopic analysis and showed focal detachment of the surface umbrella cells.
(PPTX)

S3 Fig Effects of 300 ppm BBN pretreatment and/or 10 ppm arsenic on bladder morphology. After fixation, the other half of the bladder was embedded in paraffin, cut into tissue slides, stained by H&E, and examined under an optic microscope at 400× magnification.
(PPTX)
