# Supplementary material for: The interaction of arsenic and N-butyl-N-(4-hydroxybutyl)nitrosamine on urothelial carcinogenesis in mice
Source: PLoS One. 2017 Oct 10;12(10):e0186214. doi: 10.1371/journal.pone.0186214 (PMC5634628; doi:10.1371/journal.pone.0186214)
Supplement: S1 Fig — There were 4 female mice in each group, 2 for protein analysis and 2 for morphology analysis. The bladder tissues were homogenized in protein lysis buffer and proteins were extracted for Western blot (2 mice/group) (PPTX) [file pone.0186214.s001.pptx]

## Slide 1
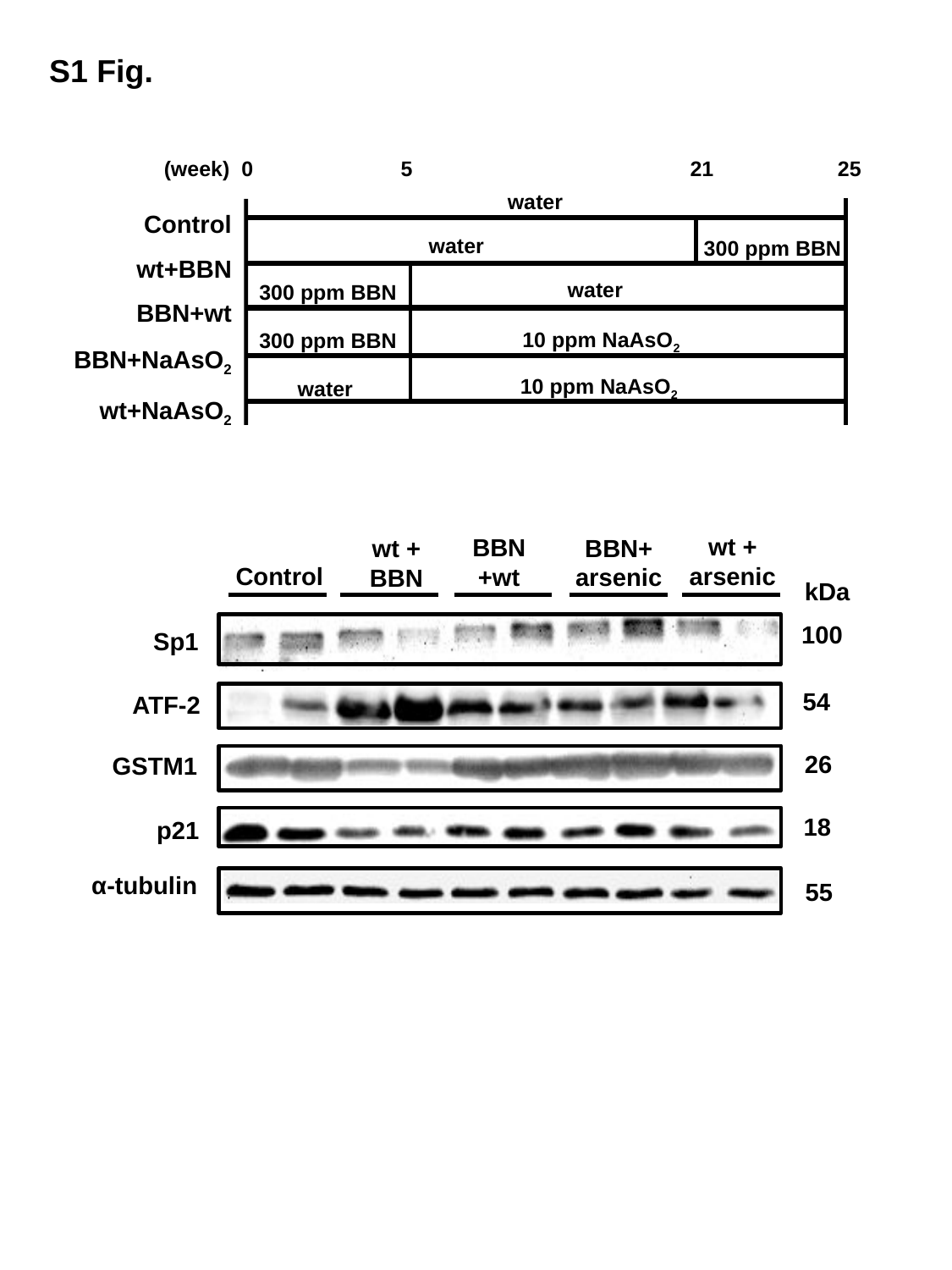

S1 Fig.
(week) 0 5 21 25
water
Control
wt+BBN
BBN+wt
BBN+NaAsO2
wt+NaAsO2
water
300 ppm BBN
water
300 ppm BBN
10 ppm NaAsO2
300 ppm BBN
10 ppm NaAsO2
water
wt +
arsenic
BBN
+wt
BBN+ arsenic
wt +
BBN
Control
kDa
100
Sp1
54
ATF-2
26
GSTM1
18
p21
α-tubulin
55
