# Supplementary figures and images for: The interaction of arsenic and N-butyl-N-(4-hydroxybutyl)nitrosamine on urothelial carcinogenesis in mice
Source: PLoS One. 2017 Oct 10;12(10):e0186214. doi: 10.1371/journal.pone.0186214 (PMC5634628; doi:10.1371/journal.pone.0186214)

## Slide 1
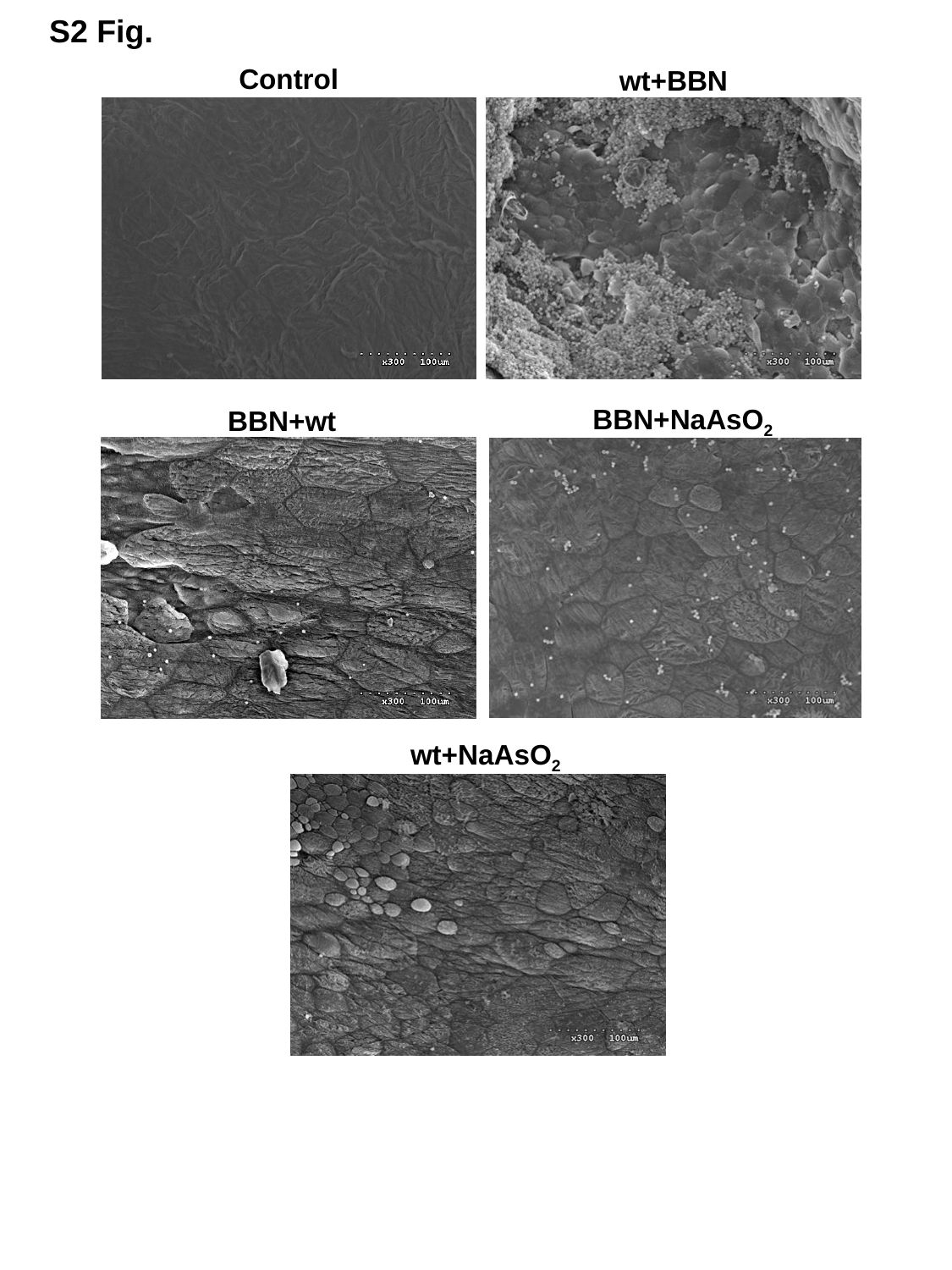

S2 Fig.
Control
wt+BBN
BBN+NaAsO2
BBN+wt
wt+NaAsO2

Supplement: S2 Fig — Bladder tissues (2 in each group) were filled with fixation buffer and then cut into two halves. One-half was prepared for scanning electron microscopic analysis and showed focal detachment of the surface umbrella cells. (PPTX) [file pone.0186214.s002.pptx]

## Slide 1
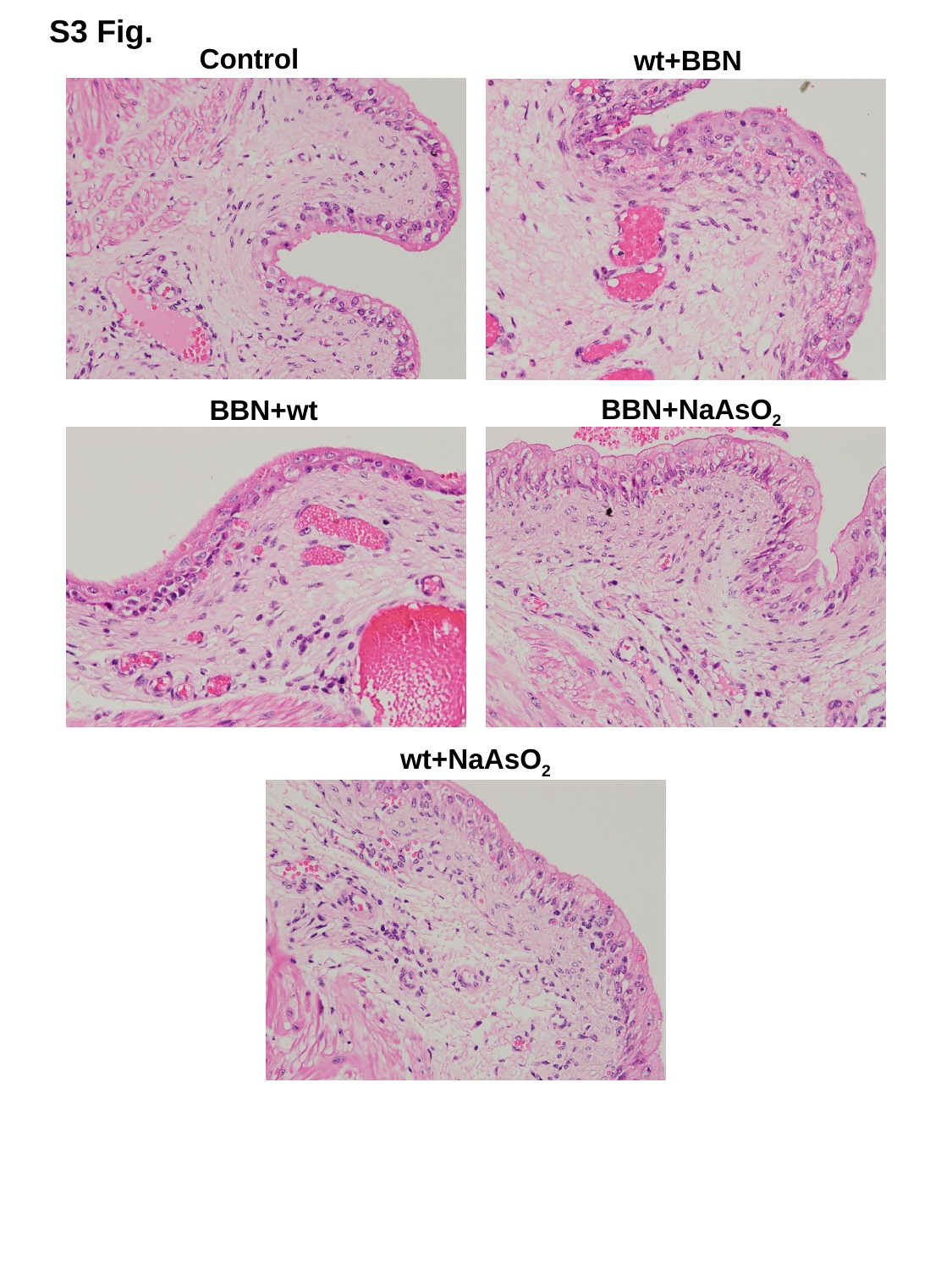

S3 Fig.
Control
wt+BBN
BBN+NaAsO2
BBN+wt
wt+NaAsO2

Supplement: S3 Fig — After fixation, the other half of the bladder was embedded in paraffin, cut into tissue slides, stained by H&E, and examined under an optic microscope at 400× magnification. (PPTX) [file pone.0186214.s003.pptx]
